# Intermittent supplementation with rapamycin as a dietary restriction mimetic

**DOI:** 10.18632/aging.100401

**Published:** 2011-12-05

**Authors:** Valter D Longo, Luigi Fontana

**Affiliations:** ^1^ Andrus Gerontology Center, University of Southern California, Los Angeles, CA, USA; ^2^ Department of Internal Medicine, Washington University School of Medicine, St. Louis, Missouri; ^3^ Division of Nutrition and Aging, Istituto Superiore di Sanitrà, Rome, Italy

Aging is a complex process associated with accumulation of damage, loss of function and increased vulnerability to disease, leading ultimately to death. Despite the complicated etiology of aging, an important discovery of recent years has been that simple genetic alterations can cause a substantial increase in healthy lifespan in laboratory model organisms [1]. Many of these longevity-extending mutations down-regulate the activity of the mTOR/S6K pathway [2-5] suggesting that reduced Tor/S6K signaling promotes entry into alternative phases normally entered during periods of starvation. In fact, dietary restriction (DR), a reduction in food intake without malnutrition, lowers Tor/S6K signaling and extends the average and maximum life span of a variety of organisms including yeast, flies, worms, fish, and rodents [1]. In both rodents and monkeys, it delays loss of function and reduces the incidence of major diseases [1] and in humans it causes a reduction in several metabolic factors and markers of diseases associated with including diabetes, and cardiovascular disease and cancer [6]. In yeast, down-regulation of the Tor/S6 pathway was shown to be important for the effects of DR on longevity and stress resistance [7].

Recently, it has been demonstrated that supplementation with rapamycin (an inhibitor of mTOR) started both at 9 and 20 months of life determines a small but significant extension of average and maximal life span in genetically heterogeneous male and female mice [8, 9]. However, in these studies rapamycin supplementation did not change the distribution of causes of death, and in particular did not reduced cancer. Interestingly, in recent issue of *Cell Cycle* Anisimov and et al. reported that lifelong intermittent administration (three times a week for 2 weeks, followed by a 2 week break) of rapamycin started at 2 mos of age significantly increased maximal lifespan and delayed spontaneous cancer in normal inbread female 129/Sv mice [10]. This study is in agreement with the effect of inhibition of Tor/S6K signaling in protecting yeast against age-dependent DNA mutations [11] and of mutations in GH or GHR in reducing cancer incidence in mice and humans [1, 12]. Similarly, DR without malnutrition extends lifespan and powerfully protects against cancer in mice [13].

Rapamycin is an immunosuppressant and antiproliferative agent that is clinically used to prevent rejection in organ transplantation, primarily in renal transplant patients. Rapamycin by inhibiting the activation of mTORC1, inhibits effector T-cell proliferation and dendritic cell maturation, but does not supress T_Reg_ cells induction [14]. Data from experimental studies indicate that rapamacyin prolongs allograft survival and reverses acute rejection of kidney allografts in in rodents and humans [15]. Besides the well-known immunosuppressive and anti-rejection properties of rapamycin, there is accumulating scientific evidence supporting a potential anti-atherogenic, anti-fibrotic, antiangiogenic, and anticancer effect of rapamycin [16]. Nevertheless, chronic subministration of rapamycin is associated with a number of side effects in some renal transplant patients, including impaired wound-healing, lymphoceles, delayed graft function, anemia, pneumonitis, hypercholesterolemia and proteinuria [15, 16]. However, these side effects are dose-dependent, and it is not known if lower doses and intermittent subministration may limit some of these effects and potentiate the beneficial effects. Interestingly in this paper rapamycin was able to extend lifespan and reduce cancer risk in mice also when used with an intermittent schedule. More studies are needed to understand benefits and side-effects of rapamycin supplementation in different strains of mice and in monkeys as a candidate cancer-preventive and life-extension pharmacological agent. However, the efficacy of intermittent rapamycin treatment in cancer prevention and life span extension shown by Anisimov et al. is very promising since it is likely to reduce the side effects associated with chronic treatment.
